# Foods to Prevent Cardiometabolic Disease: Moving from “Saturated Fat” to “Whole Foods”

**DOI:** 10.1016/j.cdnut.2026.107717

**Published:** 2026-05-16

**Authors:** Arne Astrup, Martina Abodi, Alessandra Mazzocchi, Carlo Agostoni, James Thomas Brenna, Ronald M Krauss, James O Hill, Robert H Eckel

**Affiliations:** 1 Clinical Nutrition Unit, Copenhagen University Hospital – Bispebjerg and Frederiksberg, Copenhagen, Denmark; 2 Department of Clinical Sciences and Community Health, Dipartimento di Eccellenza 2023-2027, University of Milan, Milano, Italy; 3 Infertility Unit, IRCCS Fondazione Ca’ Granda Ospedale Maggiore Policlinico, Milano, Italy; 4 Pediatric Unit, IRCCS Fondazione Ca’ Granda Ospedale Maggiore Policlinico, Milano, Italy; 5 Departments of Pediatrics, of Chemistry, and of Nutrition, Dell Pediatric Research Institute, University of Texas at Austin, TX, United States; 6 Departments of Pediatrics and Medicine, University of California, San Francisco, San Francisco, CA, United States; 7 Department of Nutrition Sciences, University of Alabama at Birmingham, AL, United States; 8 Department of Medicine, University of Colorado Anschutz Medical Campus, Aurora, Colorado, United States

**Keywords:** saturated fatty acids, cardiometabolic diseases, cardiovascular disease, dietary guidelines, dietary recommendations, palmitic acid

## Abstract

Diet and lifestyle offer the potential for achieving a healthy lifespan by reducing risk for cardiometabolic diseases and cancer, as well as maintaining cognitive health and physical function. To facilitate translation and implementation of dietary practices, we have critically reviewed the justification of the current focus on single nutrients, most notably a 10% cap for dietary saturated fat intake and argue for a more holistic food-based approach. In the newly released Dietary Guidelines for Americans, 2025–2030, the recommendation for selecting low-fat dairy foods has been changed to allow full-fat dairy food consumption in recognition of the evidence for their health benefits despite their high content of saturated fat. However, the 10% cap on total saturated fat intake has been maintained, which would require reduced saturated fat consumption from other food sources. Our analysis shows that there is considerable heterogeneity in the physiological and health effects of individual saturated fatty acids, and that the health effects of foods are determined by the food matrix and the overall nutrient profile rather than their saturated fatty acid content. We therefore conclude that the 10% cap on saturated fat intake should be replaced by food-based dietary guidelines.

## Introduction

Diet is crucial in preventing cardiometabolic diseases (CMD), defined as obesity, type 2 diabetes (T2D), and cardiovascular disease (CVD), which now more broadly includes chronic kidney disease [1] and metabolic dysfunction-associated steatotic liver disease. Since the 1950s, dietary guidelines have aimed to prevent and manage these conditions. Ancel Keys' observational study suggested that high intakes of dietary saturated fats (SFAs) and cholesterol were major causes of CVD in the United States, leading to the widely accepted "diet-heart hypothesis” [2]. Since the introduction of the United States Dietary Guidelines for Americans (DGA) in 1980, limiting SFA intake has been a central dogma for reducing atherosclerotic CVD, as endorsed by the American Heart Association, European Union, Nordic countries, and the WHO [3]. This recommendation remained in the 2025 DGA [4].

### History of saturated fat recommendations

Since 1980, an expanding body of evidence has raised questions about the validity of the original observations underpinning the diet–heart hypothesis. Systematic reviews and meta-analyses of both observational studies and randomized controlled trials (RCTs) have not provided consistent, convincing support for recommendations to reduce total SFAs intake [5,6]. Moreover, biochemical and physiological studies in humans have also provided insight into the health effects of the different SFAs, the role of the food matrix for CMD impact, and the limitations of traditional biomarkers used to assess and monitor CVD risk in response to dietary changes.

Against this background, the continued 2025 DGA recommendation to cap saturated fat intake at 10% of total energy intake warrants critical reappraisal. Although the DGA applies to all nutrition programs sponsored by the United States government, the United States passed the “Whole Milk for Healthy Kids” Act (signed into law 14 January, 2026 [7]) expressly permitting serving of full-fat milk in schools and exempts saturated fat from fluid milk from the 10% limit. Nevertheless, the 10% limit applies broadly to all other nutrition programs. The present narrative review examines the key arguments and evidence underlying this quantitative target. Specifically, we evaluate whether treating SFAs as a homogeneous group is scientifically justified, and we assess the health effects of individual SFAs within the context of the food matrices in which they naturally occur. Finally, we outline research priorities necessary to inform future dietary recommendations for CVD prevention and to guide the development and validation of more informative biomarkers for monitoring changes in CVD risk.

### Evidence from RCT and observational studies on saturated fat and CVD

“Synthesizing the evidence presented below, it is clear that even meta-analyses of RCTs, the highest tier of evidence, reach heterogeneous conclusions regarding the effects of reducing total dietary SFA on CVD outcomes.” Importantly, these analyses typically rest on the implicit assumption that individual SFAs exert broadly similar biological effects and that the food matrices in which they are consumed have minimal influence on the clinical endpoints assessed.

As evidence to justify the recommendations to reduce SFA intake, the WHO 2018 Guidelines used a Cochrane meta-analysis from 2015 that included data from 15 RCTs to assess the effect of reducing SFA on 12 hard endpoints, of which 3 were primary endpoints: *1*) all-cause mortality (deaths from any cause), *2*) CVD mortality (deaths from myocardial infarction, stroke, and/or sudden death), *3*) combined CVD events, and the remaining secondary endpoints [8]. From this analysis, it was concluded that a reduction in SFA decreased the composite endpoint of CVD events by 17% [relative risk (RR) 0.83; 95% confidence interval (CI): 0.72, 0.96], with no significant effect on the 2 other primary endpoint or any of the other hard endpoints, that is, there was no significant effect from reducing SFA intake on *1*) total mortality, *2*) CVD mortality, *3*) coronary artery disease (CAD) mortality, *4*) fatal heart event, *5*) nonfatal heart event, *6*) CAD events, nor *7*) strokes. Notably, in sensitivity analyses restricted to trials that successfully achieved meaningful reductions in SFA intake, the association with CVD events was no longer statistically significant. In an updated meta-analysis from 2020, Hooper et al. [9] reported very similar results with the only significant endpoint among the 8 being tested was the composite CVD endpoint. However, sensitivity analysis omitting the trials which included dietary interventions in addition to changes to dietary fat (e.g., changes in intake of fruit, vegetable, or fiber), also made this endpoint insignificant [RR 0.86 (95% CI: 0.67, 1.09)].

Thus, the evidence did not demonstrate any consistent benefits across hard clinical endpoints, a limitation that was highlighted shortly after publication [10].

In 2022, subsequent WHO recommendations were informed largely by observational evidence with a meta-analysis of 112 publications including 3,696,568 participants. It concluded that reducing SFA intake and replacing it with other nutrients supported the recommendation to limit SFAs consumption [11]. However, among 22 reported analyses examining total dietary SFAs, dairy SFAs, animal SFAs, circulating SFAs, and circulating palmitic acid (C16:0) in relation to cardiometabolic outcomes (Table 1) [11], only 5 associations reached statistical significance. Four of these involved circulating or tissue SFAs, particularly C16:0 (Table 1). However, circulating and adipose tissue SFAs and particularly C16:0 are not strong markers of dietary intakes of these SFAs as a substantial proportion of the circulating fatty acids (FAs) are not derived from dietary fat but are synthesized endogenously by de novo lipogenesis (DNL) that is promoted by dietary carbohydrates and alcohol, and further stimulated by weight gain, overweight, and obesity [12]. C16:0 is the most abundant SFAs in the body, accounting for roughly 20% to 30% of FAs in membrane phospholipids and adipose triacylglycerols, with levels normally kept within a narrow range by metabolic regulation. However, excessive tissue accumulation may occur under conditions of high carbohydrate intake, positive energy balance, weight gain, and low physical activity [13]. In conclusion, these analyses (Table 1) do not support that a reduction of dietary SFAs provides any CMD benefit and may instead raise the possibility that the current recommendations could have a harmful impact if people replace SFA-rich foods with carbohydrate-rich foods deprived of fiber and whole grains.

**TABLE 1:** Summary of evidence on dietary and biomarker-derived SFAs in relation to major cardiometabolic outcomes.

Data source	Exposure	Cases	People	RR (95% CI)	Grade	Comments
All-cause mortality						
Dietary intake	Total SFA	213,579	1,211,729	1.08 (1.00, 1.17)	Low	Not significant
Dietary intake	>10 vs. <10% TE SFA	194,456	1,095,528	**1.09 (1.01**, **1.18)**	Low	Confounded by other dietary intakes
Dietary intake	Animal SFA	724	17,530	1.05 (0.82, 1.35)	Very low	Not significant
Dietary intake	Dairy SFA	6982	137,739	1.05 (0.64, 1.71)	Very low	Not significant
Tissue measurements	C16:0	3832	4722	1.18 (0.91, 1.53)	Very low	Not significant
CAD Incidence (fatal and nonfatal)						
Dietary intake	Total SFA	19,263	570,326	1.04 (0.98, 1.12)	Low	Not significant
Dietary intake	>10 vs. <10%TE SFA	10,538	268,221	1.00 (0.87, 1.14)	Low	Not significant
Dietary intake	Animal SFA	3509	85,917	1.06 (0.96, 1.17)	Very low	Not significant
Dietary intake	Dairy SFA	3464	75,425	1.00 (0.98, 1.02)	Very low	Not significant
Tissue sample	Total SFA	3916	24,108	**1.46 (1.09**, **1.94)**	Low	∗Confounded by DNL from CHO, etc.
Tissue measurements	C16:0	3155	8882	1.06 (0.92, 1.23)	Very low	Not significant
CVD incidence (fatal and nonfatal)						
Dietary intake	Total SFA	68,232	1,088,501	1.07 (0.99, 1.17)	Low	Not significant
Dietary intake	>10 vs. <10%TE SFA	61,329	969,859	1.10 (0.99, 1.22)	Very low	Not significant
Dietary intake	Dairy SFA	6103	137,739	0.92 (0.75, 1.12)	Low	Not significant
Tissue measurements	C16:0	2004	4722	**1.12 (1.01**, **1.25)**	Very low	∗Confounded by DNL from CHO, etc.
Ischemic stroke incidence (fatal and nonfatal)						
Dietary intake	Total SFA	6400	402,847	0.98 (0.87, 1.12)	Low	Not significant
Dietary intake	>10 vs. <10%TE SFA	3048	172,688	0.91 (0.67, 1.24)	Very low	Not significant
Tissue measurements	C16:0	699	7739	1.19 (0.93, 1.52)	Very low	Not significant
Type 2 diabetes incidence						
Dietary intake	Total SFA	15,727	351,134	1.02 (0.95, 1.10)	Low	Not significant
Dietary intake	>10 vs. <10%TE SFA	7294	118,400	1.01 (0.82, 1.23)	Very low	Not significant
Tissue sample	Total SFA	2599	18,867	**1.30 (1.06**, **1.59)**	Very low	∗Confounded by DNL from CHO, etc.
Tissue measurements	C16:0	18,094	75,922	**1.41 (1.21**, **1.64)**	Very low	∗Confounded by DNL from CHO, etc.

RRs and 95% CIs are reported for all-cause mortality, CAD, total CVD, ischemic stroke, and type 2 diabetes incidence, based on dietary intake and tissue measurements. The RRs are determined by substitution models, measuring the impact of replacing 5% of saturated fat energy with other macronutrients. The analysis also included extreme quantile comparisons (highest vs. lowest intake) and dose–response evaluations for health outcomes. The bold indicates tha these numbers are significant.

*Abbreviations*: C16:0, palmitic acid; CAD, coronary artery disease; CHO, carbohydrates; CI, confidence interval; CVD, cardiovascular disease; DNL, de novo lipogenesis; GRADE, Grading of Recommendations Assessment, Development and Evaluation; RR, relative risk; TE, total energy.

A recent high-quality overview of systematic reviews, using an a priori protocol based on guidelines for the conduct of overviews of reviews by the Cochrane Collaboration, and including quality assessment using Grading of Recommendations Assessment, Development and Evaluation and AMSTAR-2, was published in 2023 [14]. They concluded that “The systematic reviews investigating the impact of SFA on mortality and major cancer and cardiometabolic outcomes almost universally suggest very small absolute changes in risk, and the data are based primarily on low and very low certainty evidence,” and “Our findings show considerable uncertainty around linking SFA reduction with improved health outcomes. These findings are contrary to the orthodox position of the nutrition community wherein the majority of national nutrition guidelines promote reducing or replacing SFA intake for the general population” [14].

In conclusion, neither the WHO reviews nor the most recent and most rigorously conducted analyses of this topic provide robust evidence to support that a reduction in SFAs, when viewed as a homogeneous group, will lead to a reduction in CMD.

### Effects of individual SFAs on CMD

An obvious reason for the lack of clear associations between SFAs intake and health outcomes could be the heterogeneity of SFAs making up the group, so that individual SFAs might possess positive, neutral or adverse effects (Table 2) [15]. Consequently, it is relevant to review the physiological effects of the individual SFA. Traditionally, SFAs are classified according to their carbon chain length, which provides a useful framework for discussing their distinct biological properties.

**TABLE 2:** Major naturally occurring SFAs.

Abbreviation	Common or systematic name	Carbon chain length	Major dietary sources	Overall cardiometabolic effect (positive +/neutral/negative ÷)
4:0	Butyric	Short	Dairy foods	+ +
6:0	Caproic	Short	Dairy foods	
8:0	Caprylic	Medium	Dairy foods, coconut, and palm kernel oils	+ +
10:0	Capric	Medium	Dairy foods	+ +
12:0	Lauric	Medium	Coconut milk and oil	+/neutral
14:0	Myristic	Long	Dairy foods	+/neutral/–
15:0	Pentadecanoic	Long	Red meat, dairy foods, oils	+/neutral
16:0	Palmitic	Long	Red meat, dairy foods, and palm oil	—
17:0	Heptadecanoic	Long	Red meat, dairy foods	+/neutral
18:0	Stearic	Long	Dairy foods, meat, and chocolate	+/neutral

C15:0 and C17:0 are predominantly obtained from food sources, whereas circulating levels of all other SFA are influenced by both dietary intake and endogenous metabolism. Elaborated from data reported in Perna et al. [15].

#### Short-chain, and odd and branched-chain SFAs

The odd- and branched-chain fatty acids (OBCFAs) in milk from cows' and other ruminants typically represent 2.0% to 9.5% of all dietary FAs depending on factors like feeding system, feed composition, etc., which influence the rumen microbiota composition [[16], [17], [18]]. In yak, OBCFAs can reach values close to the upper range of this spectrum (≤9.5%), highlighting that natural milks can contain relatively high levels of these FAs [18]. Milk fat is one of the most complex natural sources of FAs with around 400 different FAs with chain length from C2 to C28, including both even- and odd-numbered SFAs, MUFAs, PUFAs cis and trans, linear and branched, and various keto- and hydroxy-FAs [16], with the short- and medium-chain SFAs being C4 to C12 (Table 1). These FAs are synthesized by DNL as well as from microbial metabolism in the rumen. Most short-chain FAs are produced by human microbiota, mainly acetate, propionate, and butyrate. Full-fat dairy also provides butyrate, caproic, and caprylic acids. There is accumulating evidence to support that these FAs possess positive effects on peripheral metabolism and may contribute to reduced risk of T2D and CMD and exert positive effects on brain metabolism, that is, satiety and cognition [15,[19], [20], [21], [22]].

#### Medium-chain SFAs

Dietary intakes of the medium-chain FAs lauric acid (C12:0) mainly stem from coconut oil [23]. Observational studies using serum-free FAs measurement as biomarkers of intake of 12:0 found that the intake was negatively associated with CAD mortality [hazard ratio (HR): 0.76; 95% CI: 0.59, 0.98] [24]. The biological properties of medium-chain SFAs, such as capric and C12:0, include promotion of ketone body formation and a resulting neuroprotection that reduces the risk of dementia [25], but also reduction of insulin resistance and lowered risk of T2D [26]. A recent meta-analysis of RCTs on the effects of coconut oil on lipid profiles found varying effects on total-cholesterol and LDL cholesterol, but increased HDL cholesterol and decreased triglycerides [[26], [27], [28]]. In another meta-analysis, effects on body composition and weight loss were retrieved on the substitution of long-chain triglycerides with medium-chain triglycerides [29].

#### Long-chain SFAs

C16:0 is the most abundant SFA in most Western diets, accounting for ∼50% to 60% of total dietary SFAs intake [12]. Despite its predominance, evidence of linking C16:0 intake to metabolic outcomes is not entirely consistent. In a recent systematic review and meta-analysis, higher intakes of the long-chain SFAs C16:0 and stearic acid (C18:0) were not associated with the risk of T2D when the highest intake was compared with the lowest intake category [26]. In contrast, shorter-chain SFAs showed inverse associations. The risk of T2D decreased by 11% in the highest compared with the lowest category of dietary C12:0 (HR = 0.89; 95% CI: 0.82, 0.97), and by 17% in the highest compared with the lowest category of dietary myristic acid (C14:0) (HR = 0.83; 95% CI: 0.74, 0.92) [26]. Overall, these findings do not support a significant association between total dietary SFA intake and T2D risk.

When considering cardiovascular outcomes, both observational studies and RCTs indicate that C16:0 from both exogenous and endogenous sources is adversely associated with CVD risk, as well as total mortality [12]. In contrast, C18:0 appears to exert distinct biological effects on CVD risk markers and mediators and is generally found to be neutral for T2D and CVD risk [11].

Beyond even-chain SFA, increasing attention has been given to odd-chain FAs. Pentadecanoic acid (C15:0) and heptadecanoic acid (C17:0), predominantly found in dairy products but also present in meat and certain oils, are not synthesized endogenously in meaningful amounts. Consequently, their circulating concentrations are widely used as biomarkers of intake. Large observational studies have analyzed associations between intake and risk of CMD and generally find protective or neutral effects [30]. Liang et al. [31] conducted a systematic review and meta-analysis of circulating levels of C15:0, C17:0, and another diary-derived FA, trans-palmitoleic acid (trans-16:1n-7) and CVD risk, which included CAD, stroke, heart failure, and CVD mortality. They found the risk of CVD for the top third compared with bottom third C15:0, C17:0 and trans-16:1n-7 level was 0.94 (95% CI: 0.77, –1.15), 0.82 (95% CI: 0.68, 0.99), and 0.82 (95% CI: 0.67, 1.02), respectively. No signals for increased risk were detected. More recent studies have largely confirmed these findings, indicating that higher circulating levels of C15:0 and C17:0 are associated with a lower risk of T2D and CVD [[32], [33], [34]].

In conclusion, individual dietary SFAs exert very different effects on CMD. The effects on CMD of each individual FA have not been studied thoroughly in humans, as many studies have used natural foods containing mixtures of different FAs. However, the totality of evidence points to a very heterogeneous group of FAs in terms of CMD effects, in that short- and medium-chain SFA and some long-chain SFA have beneficial effects on CMD, and only C16:0 seem to have a negative effect on CMD risk. The potential adverse effect of C16:0, however, may be due to confounding by accompanying components in the foods, for example, processing of meat and from endogenous production from increased carbohydrate consumption. SFAs should not be viewed as a homogeneous group when it comes to effects on cardiometabolic health.

### Is the food matrix more important than the SFA content?

There is increasing recognition that other nutrient components of foods and their mutual interactions together with the food matrix are equally important as, or may even override, any effect of SFAs on CVD risk factors. Moreover, many foods we generally recognize as healthy contain SFAs, for example, olive oil, which has twice the amount of SFAs than an equivalent amount of ice cream, indicating that various foods contribute to daily SFA intake. Dietary guidelines typically highlight dairy and meat as significant sources of SFAs, so it is relevant to consider the FA composition of these and other foods when assessing their health effects. Below, we review some of the important contributing food sources of SFAs, such as whole-fat dairy, dark chocolate, and unprocessed meat.

#### Chocolate

Cocoa contains an ample amount of SFA C18:0, but also numerous other nutrients and components that have been suggested to have beneficial effects on CMD, cognition, and mood. When mixed with cream and sugar to produce chocolate, the health effects may be altered. By contrast, numerous studies have found that chocolate intake reduces risk factors of CMD [35]. Chen et al. [35] conducted a meta-analysis of RCTs, and found that cocoa products significantly decreased LDL cholesterol, triglycerides, blood glucose, and inflammation (C-reactive protein). These short-term effects are supported to be sustained by observational cohort studies [36]. A meta-analysis of prospective studies [14 publications (23 studies including 405,304 participants and 35,093 cases of CVD)] showed that chocolate consumption may be associated with reduced risk of CVD at <100 g/wk consumption [relative risk (RR) per 20 g/wk increase in chocolate consumption was 0.982 (95% CI: 0.972, 0.992; I^2^ = 50.4%; *n* = 18)] for CVD [heart failure: 0.995 (0.981–1.010, I^2^ = 36.3%, *n* = 5); total stroke: 0.956 (0.932–0.980, I^2^ = 25.5%, *n* = 7); cerebral infarction: 0.952 (0.917–0.988, I^2^ = 0.0%, *n* = 4); hemorrhagic stroke: 0.931 (0.871–0.994; I^2^ = 0.0%; *n* = 4); myocardial infarction: 0.981 (0.964–0.997, I^2^ = 0.0%, *n* = 3); CAD: 0.986 (0.973–0.999, *n* = 1)] [37]. It is noteworthy that the systematic reviews and meta-analyses cited above were funded by public research foundations and government institutions.

#### Dairy

Dairy products all originate from animal milk but constitute a heterogenous group of foods that vary in physical state and structure, nutrient profiles (including bioactive ingredients and microorganisms) and level and type of processing (e.g., fermented, aged, condensed). Dairy accounts for ∼30 % of SFAs intake in the United States [38], and cheese makes up ∼23% of SFAs, with the remainder due to milk, yoghurt, ice cream, and butter. Dairy foods contribute substantially to the intake of essential nutrients to the diet, such as high-quality protein, calcium, vitamin D, potassium, and other vitamins and minerals. Moreover, dairy foods are important sources of bioactive compounds, including dairy-derived FAs, phospholipids, peptides, and the milk fat globule membrane [39]. Although milk with its liquid matrix is the primary ingredient of all other dairy foods, the nutrient and bioactive composition and physical structure of milk shift markedly during processing to yogurt (gel matrix) and cheese (semisolid or solid matrix).

Epidemiological evidence suggests that the health effects of dairy products may differ according to specific subtypes. For example, analyses from large United States prospective cohorts reported heterogeneous associations with cardiometabolic outcomes: yogurt consumption was inversely associated with T2D risk (HR ≈0.83 per serving/d), whereas higher intakes of cheese and whole milk showed modest positive associations in some analyses [40].

Evidence from RCTs synthesized in a recent network meta-analysis also suggests that dairy foods may have heterogeneous effects on cardiometabolic markers [41]. Both low-fat and full-fat dairy consumption were associated with reductions in systolic blood pressure (approximately −5 to −8 mmHg), whereas full-fat dairy increased HDL cholesterol. In addition, yogurt showed more favorable effects compared with milk, including reductions in waist circumference and triglycerides and increases in HDL cholesterol. Overall, the analysis concluded that higher dairy intake is unlikely to have detrimental effects on cardiometabolic health markers [41].

Evidence from substitution analyses in prospective cohort studies further indicates that replacing dairy products with other food groups may differentially influence cardiometabolic risk [42]. In particular, substituting low-fat dairy with red or processed meat was associated with higher risks of mortality, CAD, and T2D, whereas replacing dairy or yogurt with whole grains and butter with olive oil was associated with lower mortality risk. In contrast, substituting dairy products with other dairy foods generally showed neutral associations with disease risk [42].

Moreover, evidence from recent systematic reviews and meta-analyses indicates that fermented dairy products may be associated with favorable major cardiovascular events. Synthesis of the evidence from prospective cohort studies suggests that fermented dairy intake, including yogurt and cheese, is associated with lower CVD risk, with pooled estimates indicating reductions in CVD risk of ∼13% for cheese and 22% for yogurt in some analyses [37]. Overall, these findings support the hypothesis that cardiometabolic effects of dairy foods may vary according to the specific dairy subtype and processing characteristics, particularly fermentation [43]. In a recent global analysis combining the United Kingdom Biobank and China Kadoorie Biobank together with an updated meta-analysis of prospective cohorts, total dairy consumption was associated with a modestly lower risk of CVD (with approximately a 2% lower risk of for each additional serving/d of total dairy intake) and stroke (RR 0.94; 95% CI: 0.90, 0.98), with no differences in the risk of CAD. Instead, cheese consumption, in particular, was inversely associated with incident CVD and CAD. Fermented dairy products, especially cheese, were associated with a reduced CVD risk (RR 0.96; 95% CI: 0.94, 0.98, for fermented dairy and 0.94; 95% CI: 0.91, 0.97, for cheese), supporting the hypothesis that the health effects of dairy intake may depend on the food matrix and fermentation processes rather than SFAs content alone [43].

Except for 1 study [39], which was industry-sponsored and coauthored by dairy council representatives, the cited studies [38,[40], [41], [42], [43]] were publicly funded with no declared conflicts of interest.

#### Unprocessed meat

The relationship between meat consumption and CVD in meta-analyses indicates that the association between saturated fat intake and CVD is weaker than previously believed and depends heavily on the replacement nutrient (e.g., polyunsaturated fat compared with refined carbohydrate) [8,44], and is more complex than the traditional view that “fatty meat increases heart disease risk.” Contemporary research suggests that both SFAs content and the food matrix of meat influence cardiometabolic outcomes, yet neither factor alone fully explains the observed associations.

Red and processed meats contain varying amounts of saturated fat, which can raise LDL cholesterol, and this historically has led to the assumption that red meat directly increases CVD risk [45]. However, an RCT has demonstrated that although red meat increased LDL cholesterol compared with the same amount of nonmeat protein, the effect was relatively small and was the same as white meat intake [46]. Moreover, several large cohort studies and meta-analyses report weak or nonsignificant associations between unprocessed red meat and CVD [44,47]. These findings suggest that saturated fat from meat may not exert the same effect as isolated saturated fat, and that the broader dietary context plays a substantial role. The food matrix of meat modulates digestion, nutrient interactions, and metabolic responses. In meat, protein, fat, micronutrients, connective tissue, and bioactive compounds interact in ways that influence cardiometabolic effects. Unprocessed red meat often shows weaker associations with CVD than expected based solely on its SFAs content [47,48]. Processed meats consistently show stronger associations with CVD, likely due to nitrites, and heat-induced compounds rather than saturated fat [5]. Research comparing whole foods suggests that saturated fat from dairy foods and meat behaves differently from isolated SFAs, again highlighting the importance of the matrix [49]. These findings do not imply that high intakes of fatty meat have neutral health effects, but they underscore that the relationship is not linear and cannot be reduced to a single nutrient. In conclusion, current evidence suggests that the cardiovascular impact of meat is shaped by a combination of SFAs, processing, food matrix effects, and overall dietary patterns. Associations between unprocessed red meat and CVD have been weak or nonsignificant [44,47]. These findings suggest that SFAs from meat may not exert the same effect as isolated SFAs, and that the broader dietary context plays a substantial role. Meta-analyses indicate that, as with other sources of dietary SFAs, the association between saturated fat intake and CVD is weaker than previously believed and depends heavily on the replacement nutrient (e.g., polyunsaturated fat compared with refined carbohydrate) [8,44]. The meat-related evidence presented [[44], [45], [46], [47], [48]] primarily relies on public and institutional funding, with the exception of study 48, which received support from the beef industry. Additionally, minor industry advisory ties were noted in study [44], whereas studies [[45], [46], [47]] remain independently funded by public health organizations.

#### Fats and oils

While the food matrix modulates the cardiometabolic effects of many foods, the relatively simple matrix of most oils and fats has led to the assumption that their FAs composition can be used to predict CMD risk. However, fats and oils have various nutrients and bioactive ingredients that can have effects that override their SFAs content. The most SFA-rich fat is butter, and meta-analysis of observational studies points to butter intake being neutral for CVD risk and inversely associated with diabetes risk when comparing high compared with low intakes in cohort studies [50]. By contrast, olive oil and canola oil have both been shown to reduce hard CVD endpoints in RCT’s. In the Lyon Diet Heart study, a Mediterranean diet supplemented with canola oil-based margarine reduced cardiovascular mortality by 73% and overall mortality by 70% [51]. However, subsequent analyses suggested that the effect was not due to the low content of SFAs and high content of MUFAs and PUFAs but rather due to the high content of alpha-linolenic acid. In addition, robust evidence supports a protective role of olive oil consumption on hard CVD endpoints [51]. In the PREDIMED RCT, a Mediterranean diet supplemented with extravirgin olive oil significantly reduced the incidence of major cardiovascular events compared with a low-fat control diet [52]. The cardioprotective effects of olive oil seem to be due to the presence of a plethora of polyphenolic compounds, such as oleuropein, tyrosol, and hydroxytyrosol [53].

In recent years, large United States cohort studies have highlighted differential associations between specific fats and mortality [54]. After adjustment for relevant confounders, higher intakes of canola oil were associated with about a 15% lower mortality risk, whereas olive oil and soybean oil were associated with about an 8% and 6% lower mortality risk, respectively. In contrast, higher butter intake was associated with a 15% higher risk of total mortality. Importantly, replacing 10 g/d of butter with an equivalent amount of plant-based oils was associated with a 17% lower risk of total mortality [54]. These findings were derived from long-term follow-up analyses using repeatedly updated food frequency questionnaires and multivariable Cox models, supporting the importance of fat quality and substitution patterns, rather than total fat intake alone, in relation to mortality risk.

The evidence regarding plant oils and butter [[50], [51], [52], [53], [54]] is primarily based on public and institutional funding. However, industry involvement was noted in studies [51,52], which received financial support or food products from companies related to vegetable oils and nuts. Studies [50,53,54] were independently funded with no declared conflicts of interest.

In conclusion, our analysis shows that there is considerable heterogeneity in the physiological and health effects of individual SFAs, and that the health effects of foods are determined by the food matrix and the overall nutrient profile rather than by their SFAs content alone. We therefore conclude that the current 10% cap on SFAs intake should be replaced by food-based dietary guidelines. In addition, nutrition research should move away from continuing to publish studies that treat SFAs as a single homogeneous entity and, at a minimum, consistently incorporate valid biomarkers of individual FAs alongside food-based analyses. Furthermore, there is a clear need for more studies that directly examine the effects of whole foods containing SFAs on hard CVD endpoints, rather than continuing to assume adverse health effects solely based on their SFAs content.

## Author contributions

The authors’ responsibilities were as follows – AA: conceived the research, conducted the literature review, and wrote the first draft of the manuscript; MA, AM: critically reviewed and revised the manuscript for important intellectual content; CA, JTB, RMK, JOH, RHE: contributed to the interpretation of the evidence and critically reviewed the manuscript to ensure scientific accuracy and integrity; RHE: primary responsible for final content; and all authors: read and approved the final manuscript.

## Declaration of generative AI and AI-assisted technologies in the writing process

The author(s) declare that no generative AI or AI-assieted technologies were used in the writing of this manuscript, apart from checking for potential missing studies and references.

## Data availability

Data described in the manuscript will be made available upon request.

## Funding

The article is based on a Symposium “One Quality, One Health” held at the 11th International Conference on Nutrition and Growth (Lisbon, 15 February, 2024), supported by Soremartec Italia S.r.l. Its contents are solely the responsibility of the authors.

## Conflict of interest

The authors report no conflicts of interest.

## References

[1] Ndumele C.E., Neeland I.J., Tuttle K.R., Chow S.L., Mathew R.O., Khan S.S. (2023). A synopsis of the evidence for the science and clinical management of cardiovascular-kidney-metabolic (CKM) syndrome: a scientific statement from the American Heart Association. Circulation.

[2] Keys A., Anderson J., Grande F. (1957). Prediction of serum-cholesterol responses of man to changes in fats in the diet. Lancet.

[3] Snetselaar L.G., de Jesus J.M., DeSilva D.M., Stoody E.E. (1980). Nutrition and your health dietary guidelines for Americans, Nutr. Today..

[4] US Department of Agriculture. Dietary Guidelines for Americans, 2025-2030 Dietary Guidelines for Americans, 2025-2030. https://cdn.realfood.gov/DGA.pdf.

[5] Yamada S., Shirai T., Inaba S., Inoue G., Torigoe M., Fukuyama N. (2025). Saturated fat restriction for cardiovascular disease prevention: a systematic review and meta-analysis of randomized controlled trials. JMA J.

[6] Shi F., Chowdhury R., Sofianopoulou E., Koulman A., Sun L., Steur M. (2025). Association of circulating fatty acids with cardiovascular disease risk: analysis of individual-level data in three large prospective cohorts and updated meta-analysis. Eur. J. Prev. Cardiol..

[7] US Department of Agriculture, Food and Nutrition Service (2026). Whole Milk for Healthy Kids Act of 2025 –Implementation Requirements for the National School Lunch Program.

[8] Hooper L., Martin N., Jimoh O.F., Kirk C., Foster E., Abdelhamid A.S. (2020). Reduction in saturated fat intake for cardiovascular disease. Cochrane Database Syst. Rev..

[9] Hooper L., Summerbell C.D., Higgins J.P., Thompson R.L., Capps N.E., Smith G.D. (2001). Dietary fat intake and prevention of cardiovascular disease: systematic review. BMJ.

[10] Astrup A., Bertram H.C., Bonjour J.P., de Groot L.C., de Oliveira Otto M.C., Feeney E.L. (2019). WHO draft guidelines on dietary saturated and trans fatty acids: time for a new approach?. BMJ.

[11] Reynolds A, Hodson L, de Souza R, Tran Diep Pham H, Vlietstra L, Mann J (2022). Saturated fat and trans-fat intakes and their replacement with other macronutrients: a systematic review and meta-analysis of prospective observational studies.

[12] Annevelink C.E., Sapp P.A., Petersen K.S., Shearer G.C., Kris-Etherton P.M. (2023). Diet-derived and diet-related endogenously produced palmitic acid: effects on metabolic regulation and cardiovascular disease risk. J. Clin. Lipidol..

[13] Carta G., Murru E., Banni S., Manca C. (2017). Palmitic acid: physiological role, metabolism and nutritional implications. Front. Physiol..

[14] Talukdar J.R., Steen J.P., Goldenberg J.Z., Zhang Q., Vernooij R.W.M., Ge L. (2023). Saturated fat, the estimated absolute risk and certainty of risk for mortality and major cancer and cardiometabolic outcomes: an overview of systematic reviews. Syst. Rev..

[15] Perna M., Hewlings S. (2022). Saturated fatty acid chain length and risk of cardiovascular disease: a systematic review. Nutrients.

[16] Kupczyński R., Pacyga K., Lewandowska K., Bednarski M., Szumny A. (2024). Milk odd- and branched-chain fatty acids as biomarkers of Rumen fermentation. Animals (Basel).

[17] Ran-Ressler R.R., Sim D., O’Donnell-Megaro A.M., Bauman D.E., Barbano D.M., Brenna J.T. (2011). Branched chain fatty acid content of United States retail cow’s milk and implications for dietary intake. Lipids.

[18] Sun W., Luo Y., Wang D.H., Kothapalli K.S.D., Brenna J.T. (2019). Branched chain fatty acid composition of yak milk and manure during full-lactation and half-lactation. Prostaglandins Leukot. Essent. Fatty Acids.

[19] Nogal A., Valdes A.M., Menni C. (2021). The role of short-chain fatty acids in the interplay between gut microbiota and diet in cardio-metabolic health. Gut Microbes.

[20] Zheng L., Jiang R., Fang J., Tan Y., Wang Y., Li L. (2025). Short-chain fatty acids and type 2 diabetes: a bibliometric analysis of knowledge structure, research hotspots, and future directions. Front. Microbiol..

[21] Brahe L.K., Astrup A., Larsen L.H. (2013). Is butyrate the link between diet, intestinal microbiota and obesity-related metabolic diseases?. Obes. Rev..

[22] Birkeland E., Gharagozlian S., Valeur J., Aas A.M. (2023). Short-chain fatty acids as a link between diet and cardiometabolic risk: a narrative review. Lipids Health Dis.

[23] Schwingshackl L., Schlesinger S. (2023). Coconut oil and cardiovascular disease risk. Curr. Atheroscler. Rep..

[24] Huang N.K., Bůžková P., Matthan N.R., Djoussé L., Hirsch C.H., Kizer J.R. (2021). Associations of serum nonesterified fatty acids with coronary heart disease mortality and nonfatal myocardial infarction: The CHS (Cardiovascular Health Study) cohort. J. Am. Heart Assoc..

[25] Hughes S.D., Kanabus M., Anderson G., Hargreaves I.P., Rutherford T., O’Donnell M. (2014). The ketogenic diet component decanoic acid increases mitochondrial citrate synthase and complex I activity in neuronal cells. J. Neurochem..

[26] Gaeini Z., Bahadoran Z., Mirmiran P. (2022). Saturated fatty acid intake and risk of type 2 diabetes: an updated systematic review and dose-response meta-analysis of cohort studies. Adv. Nutr..

[27] Hewlings S. (2020). Coconuts and health: different chain lengths of saturated fats require different consideration. J. Cardiovasc. Dev. Dis..

[28] Newport M.T., Dayrit F.M. (2025). Analysis of 26 studies of the impact of coconut oil on lipid parameters: beyond total and LDL cholesterol. Nutrients.

[29] Mumme K., Stonehouse W. (2015). Effects of medium-chain triglycerides on weight loss and body composition: a meta-analysis of randomized controlled trials. J. Acad. Nutr. Diet..

[30] Chen T., Luo J., Li S., Li X., Wang W., Lu W. (2025). Associations between serum pentadecanoic acid (C15:0) and heptadecanoic acid (C17:0) levels and hypertension: a cross-sectional analysis of NHANES data. Lipids Health Dis.

[31] Liang J., Zhou Q., Kwame Amakye W., Su Y., Zhang Z. (2018). Biomarkers of dairy fat intake and risk of cardiovascular disease: a systematic review and meta analysis of prospective studies. Crit. Rev. Food Sci. Nutr..

[32] Risérus U., Marklund M. (2017). Milk fat biomarkers and cardiometabolic disease. Curr. Opin. Lipidol..

[33] Trieu K., Bhat S., Dai Z., Leander K., Gigante B., Qian F. (2021). Biomarkers of dairy fat intake, incident cardiovascular disease, and all-cause mortality: a cohort study, systematic review, and meta-analysis. PLOS Med.

[34] Imamura F., Fretts A., Marklund M., Ardisson Korat A.V., Yang W.S., Lankinen M. (2018). Fatty acid biomarkers of dairy fat consumption and incidence of type 2 diabetes: a pooled analysis of prospective cohort studies. PLOS Med.

[35] Chen X., Guan X., Tang Y., Deng J., Zhang X. (2022). Effects of cocoa products intake on cardiometabolic biomarkers of type 2 diabetes patients: a systematic review and meta-analysis based on both long-term and short-term randomised controlled trials. Int. J. Food Sci. Nutr..

[36] Zhao B., Gan L., Yu K., Männistö S., Huang J., Albanes D. (2022). Relationship between chocolate consumption and overall and cause-specific mortality, systematic review and updated meta-analysis. Eur. J. Epidemiol..

[37] Ren Y., Liu Y., Sun X.Z., Wang B.Y., Zhao Y., Liu D.C. (2019). Chocolate consumption and risk of cardiovascular diseases: a meta-analysis of prospective studies. Heart.

[38] Wambogo E.A., Ansai N., Terry A., Fryar C., Ogden C. (2023). Dairy, meat, seafood, and plant sources of saturated fat: United States, ages two years and over, 2017-2020. J. Nutr..

[39] Unger A.L., Astrup A., Feeney E.L., Holscher H.D., Gerstein D.E., Torres-Gonzalez M. (2023). Harnessing the magic of the dairy matrix for next-level health solutions: a summary of a symposium presented at nutrition 2022. Curr. Dev. Nutr..

[40] Chen M., Sun Q., Giovannucci E., Mozaffarian D., Manson J.E., Willett W.C. (2014). Dairy consumption and risk of type 2 diabetes: 3 cohorts of US adults and an updated meta-analysis. BMC Med.

[41] Kiesswetter E., Stadelmaier J., Petropoulou M., Morze J., Grummich K., Roux I. (2023). Effects of dairy intake on markers of cardiometabolic health in adults: a systematic review with network meta-analysis. Adv. Nutr..

[42] Kiesswetter E., Neuenschwander M., Stadelmaier J., Szczerba E., Hofacker L., Sedlmaier K. (2024). Substitution of dairy products and risk of death and cardiometabolic diseases: A systematic review and meta-analysis of prospective studies. Curr. Dev. Nutr..

[43] Zhuang P., Liu X., Li Y., Ao Y., Wu Y., Ye H. (2025). A global analysis of dairy consumption and incident cardiovascular disease. Nat. Commun..

[44] Siri-Tarino P.W., Sun Q., Hu F.B., Krauss R.M. (2010). Meta-analysis of prospective cohort studies evaluating the association of saturated fat with cardiovascular disease. Am. J. Clin. Nutr..

[45] Mensink R.P. (2016). Effects of saturated fatty acids on serum lipids and lipoproteins: a systematic review and regression analysis.

[46] Bergeron N., Chiu S., Williams P.T., King S.M., Krauss R.M. (2019). Corrigendum: Effects of red meat, white meat, and nonmeat protein sources on atherogenic lipoprotein measures in the context of low compared with high saturated fat intake: a randomized controlled trial. Am. J. Clin. Nutr..

[47] Micha R., Wallace S.K., Mozaffarian D. (2010). Red and processed meat consumption and risk of incident coronary heart disease, stroke, and diabetes mellitus: a systematic review and meta-analysis. Circulation.

[48] O’Connor L.E., Kim J.E., Campbell W.W. (2017). Total red meat intake of ≥0.5 servings/d does not negatively influence cardiovascular disease risk factors: a systemically searched meta-analysis of randomized controlled trials. Am. J. Clin. Nutr..

[49] Astrup A., Magkos F., Bier D.M., Brenna J.T., de Oliveira Otto M.C., Hill J.O. (2020). Saturated fats and health: A reassessment and proposal for food-based recommendations: JACC state-of-the-art review. J. Am. Coll. Cardiol..

[50] Pimpin L., Wu J.H.Y., Haskelberg H., Del Gobbo L., Mozaffarian D. (2016). Is butter back? A systematic review and meta-analysis of butter consumption and risk of cardiovascular disease, diabetes, and total mortality. PLOS One.

[51] de Lorgeril M., Renaud S., Mamelle N., Salen P., Martin J.L., Monjaud I. (1994). Mediterranean alpha-linolenic acid-rich diet in secondary prevention of coronary heart disease. Lancet.

[52] Estruch R., Ros E., Salas-Salvadó J., Covas M.I., Corella D., Arós F. (2018). Retraction and republication: primary prevention of cardiovascular disease with a Mediterranean diet. N. Engl. J. Med..

[53] Mehmood A., Usman M., Patil P., Zhao L., Wang C. (2020). A review on management of cardiovascular diseases by olive polyphenols. Food Sci. Nutr..

[54] Zhang Y., Chadaideh K.S., Li Y., Li Y., Gu X., Liu Y. (2025). Butter and plant-based oils intake and mortality. JAMA Intern. Med..

